# In Pursuit of Work Performance: Testing the Contribution of Emotional Intelligence and Burnout

**DOI:** 10.3390/ijerph17155373

**Published:** 2020-07-26

**Authors:** Martin Sanchez-Gomez, Edgar Breso

**Affiliations:** Department of Evolutionary, Educational, Social Psychology and Methodology, Universitat Jaume I, 12071 Castellón de la Plana, Spain; breso@uji.es

**Keywords:** burnout, emotional intelligence, multioccupational sample, performance, professional efficacy, exhaustion

## Abstract

Previous research has highlighted the connection between emotional intelligence (EI) and work performance. However, the role of job burnout in this context remains relatively unexplored. This study aimed to examine the mediator role of burnout in the relationship between EI and work performance in a multioccupational sample of 1197 Spanish professionals (58.6% women). The participants completed the Wong and Law Emotional Intelligence Scale, the Maslach Burnout Inventory, and the Individual Work Performance Questionnaire. As expected, the results demonstrated a positive relationship between EI and performance, and a negative relationship with burnout, which has a mediator effect in the relationship between EI and work performance. Professionals with high levels of IE and low burnout reported the highest performance. Multiple mediation analyses showed that employees’ EI was indirectly connected to work performance via professional efficacy and exhaustion, even when controlling the effects of sociodemographic variables. The same pattern was found when multiple mediations were conducted for each EI dimension. These findings demonstrate the importance of burnout in understanding work performance and emphasize the role of EI as a protective variable which can prevent the development or chronic progression of workers’ burnout.

## 1. Introduction

### 1.1. Work Performance in the Current Situation

The current global context has created a competitive environment where companies are interested in optimizing their performance to obtain maximum profits, which depends largely on the performance of its workers [1]. Work performance has been described as behaviors or actions that are relevant to the goals of the organization [2]. Traditionally, the study of individual performance has focused on the task performance aspect, which is defined as the proficiency with which individuals perform the core substantive or technical tasks of their job [2]. However, research has since agreed to describe work performance in a way that goes beyond individual work performance to include contextual performance and counterproductive work behaviors [3,4]. Contextual performance is described as those behaviors supporting the organizational, social, and psychological environment in which the technical core must function [5]. Counterproductive work can be defined as behavior that harms the well-being of the organization [3].

In an attempt to better understand this issue, research has focused on the factors related to work performance [6]. The findings indicate that it is necessary to incorporate individual and social resources to understand the whole labor sphere, in addition to the technical competencies required to develop any profession accurately [6]. Nowadays, this relationship is particularly interesting due to the characteristics of the global work environment and the way in which organizations try to define relationships in the workplace, where reduced or nonexistent social interactions are a common theme [7]. The need to examine how different factors are related to work performance is even more relevant in Spanish companies, which find themselves in a worse financial situation than their European counterparts [8]. In this complex and everchanging context, knowing the predictors of job performance is increasingly relevant for organizations [9].

### 1.2. Emotional Intelligence and Work Performance

Previous research has proposed personal resources as fundamental variables to understand work environments [10]. Effective performance requires the conjunction of both cognitive and emotional skills that allow the individual to analyze their environment and make the best decisions [11]. Among all these variables, emotional intelligence (EI) is becoming increasingly important to understand the individual outcomes [12,13,14]. EI emerged 30 years ago [15], and since then, it has gained relevance among researchers and professionals alike [16]. According to the main model of EI proposed by Mayer and Salovey, EI is a kind of social intelligence comprising four branches, each related to a specific ability: (1) Perceiving one’s own as well as others’ feelings and emotions, (2) using emotions to facilitate thought, (3) understanding and discriminating among emotions, and (4) managing them [17].

Previous research has found that performance at work is strongly affected by emotions and feelings, which are an inherent part of the human existence in any context [18]. Those workers with higher emotional intelligence tend to be more successful [19,20], more productive [21,22], and less susceptible to perform counterproductive work behaviors [23]. Moreover, subjects with higher EI have shown more engagement than their colleagues [24], less burnout [25], and a lower intention to quit [26].

Emotional intelligence is strongly related to emotional and social skills, which positively influences skills like empathy, teamwork, communication, achievement orientation, and negotiation—all characteristics that favor good work performance [27]. For example, it has been observed that workers with higher EI can cope better with the situations that arise in their jobs and, therefore, achieve higher rates of job satisfaction, unlike those who fail to develop such capacity [27]. In addition, the relevance of self-appraisal in the service industry, where there is a high level of interaction between employees and customers, was observed [28]. Similarly, Giardini and Frese [29] noted the importance of employees generating positive emotions as a central component to customer service. Hence, emotional intelligent people are able to confront negative job events while simultaneously experiencing more psychological well-being due to their socioemotional abilities [30,31].

### 1.3. Burnout as a Mediator

It seems that several personal resources like EI are related to better performance at work, but there are also different threatening factors that could reduce the productivity [32]. Currently, a high percentage of professionals suffer from work overload, time pressure, and stressful face-to-face interactions with clients—aspects which often lead to intense chronic distress that could progress into what is known as Burnout Syndrome [33]. Burnout is defined as a prolonged response to chronic emotional and interpersonal stressors related to work. It is divided into three dimensions: Exhaustion, cynicism, and reduced professional efficacy [34]. Exhaustion refers to the feeling of not being able to give more of oneself on an emotional level. Cynicism is defined as indifference or a distant attitude toward one’s work in general. Meanwhile, professional efficacy refers to the employee’s expectations of continued effectiveness at work. The main signs and symptoms of burnout include tiredness, difficulties concentrating, poor organization, a greater number of errors, decreased quality of work, a lack of energy, anxiety, and frustration [35]. One of the most frequently cited negative consequences of burnout is a decline in job performance [36]. For example, a growing body of studies suggests that there is a strong relationship between burnout and counterproductive behaviors [37,38]. A decrease in job performance, job commitment, physical health, mental health, and an increase in job task error are examples of the consequences of work overload [39]. In conclusion, experiencing all these consequences changes how workers perceive work challenges—instead perceiving them as threats and encountering more difficulties than usual. This affects the professionals’ capacity for adaptation and can distort their performance [40].

Until a few years ago, burnout research in the workplace focused on analyzing the syndrome’s risk factors and its negative repercussions [41]. Recently, burnout has been defined as a public health problem, and new theoretical approaches argue for the study of variables, which insofar are related to burnout and can take a protective role against events that might trigger the syndrome [25]. A wide range of described factors include as social and emotional skills, communication, empathy, resilience, coping strategies, stress tolerance, a proactive personality, and self-esteem [25]. Among all these variables, EI emerges as an important and useful feature to group these skills and understand how the effects of burnout can be reduced on individual outcomes at work [42]. Several researchers have found that EI can help reduce the negative effects of the workload, exhaustion, job dissatisfaction, and stress of workers [43,44,45], contributing to improved social relations, performance, teamwork, effective leadership, etc., which have a noteworthy impact on work outcomes [45,46].

### 1.4. The Present Study

**Figure 1:**
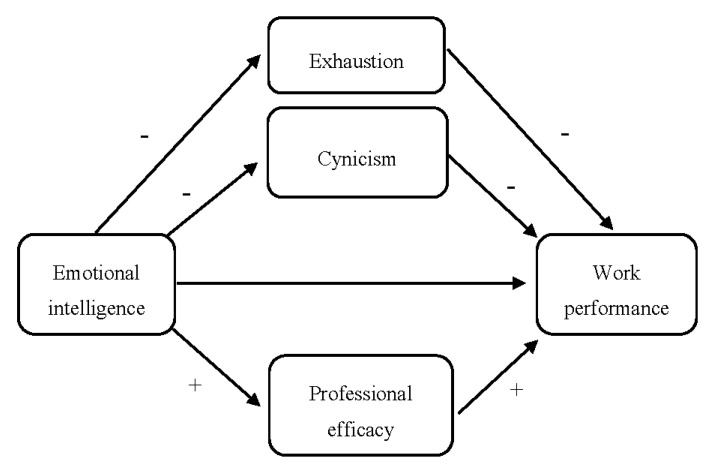
Proposed mediation model to empirically test the associations between emotional intelligence, the three burnout dimensions, and work performance.

In conclusion, research has underlined a robust link between EI and work performance, between EI and burnout, and between burnout and performance. Nevertheless, the mediator role of job burnout between EI and work performance remains relatively unexplored. Therefore, this work may help deepen the knowledge of psychosocial factors that help improve individual performance through a mediated model in which EI indirectly influences the performance through burnout (see Figure 1).

The main aim of this study was to examine the mediator role of burnout in the relationship between EI and work performance. Based on previous findings [42,45,46], it was expected that employees’ exhaustion, cynicism, and professional efficacy would mediate the relationship between EI and work performance. This hypothesis is shown in Figure 1.

## 2. Materials and Methods

### 2.1. Sample

Following a cross-sectional design, a final sample of 1197 subjects (58.6% of whom were women) was recruited. Participants belonged to different occupational sectors, such as education (29.3%), healthcare (23.5%), industry (17.1%), hospitality and tourism (15.2%), commerce (7.8%), and other sectors (7.1%).

The mean age was 37 years (M = 38.1, SD = 10.3, range = 18–64 years). The average work experience was 12 years, average organizational seniority was 8 years, and seniority in the job position was 6 years. Of the participants, 50.6% had a job at private companies, 29.3% worked in public enterprises, 11.6% were self-employed, and 8.5% were unemployed. As for the marital status of the participants, 37.9% were married, 33.7% single, 20.3% in a relationship, 5.1% separated/divorced, and 1.6% widowed. Table 1 shows the individual characteristics of the participants in terms of various significant variables.

### 2.2. Instruments

**Emotional Intelligence**. The Spanish version of the Wong and Law Emotional Intelligence Scale (WLEIS) [47] was used to evaluate the perceived EI. This adaptation of the scale has shown satisfactory psychometric properties [48]. This scale is a self-report measure made up of 16 items with a 5-point Likert-type scale. Studies on its factorial structure have found four factors with four items each: Evaluation of one’s own emotions (SEA; “I have a good sense of why I have certain feelings most of the time”), evaluation of the emotions of others (OEA; “I always know my friends’ emotions from their behavior”), use of emotions or assimilation (UOE; “I always set goals for myself and then try my best to achieve them”), and regulation of emotions (ROE; “I have good control of my own emotions”). The internal consistency of each of these branches was good: SEA (0.90), OEA (0.93), UOE (0.89), ROE (0.88). Moreover, the WLEIS offered a global score with a reliability of 0.91.

**Burnout**. This variable was measured using the Maslach Burnout Inventory (MBI-GS) [49] in its Spanish adaptation [50]. The MBI-GS Questionnaire examines the three dimensions described as parts of the burnout syndrome: exhaustion (five items; e.g., “I feel used up at the end of the workday”), cynicism (five items; e.g., “I have become less enthusiastic about my work”, and professional efficacy (six items; e.g., “In my opinion, I am good at my job”). All items were answered on a seven-point Likert scale ranging from 0 (never) to 6 (every day). The indicators of internal consistency revealed substantial internal consistency: Exhaustion (0.90), cynicism (0.86), and professional efficacy (0.79).

**Work Performance.** The Individual Work Performance Questionnaire (IWPQ) [51] was used to assess the individual work performance. A Spanish translation was developed ad hoc for this research. This 18-item scale was developed to measure the three main dimensions of job performance: Task performance (“My planning was optimal”), contextual performance (“I took on extra responsibilities”), and counterproductive work behavior (“I complained about unimportant matters at work”). All items were answered following a five-point Likert scale (0 = seldom to 4 = always for task and contextual performance; and 0 = never to 4 = often for counterproductive work behavior). Taking a previous work [52] as reference, a global score called ‘work performance’ (WP) was created to simplify the analyses. This global score is the result of calculating an average of the three variables after reversing the negative sense of the counterproductive work behavior dimension. The internal consistency was good: Task performance (0.80), contextual performance (0.85), counterproductive work behavior (0.81), and 0.88 regarding to the global score (WP).

**Control Variables**. In addition to the main study variables, more questions were introduced to obtain sociodemographic data (i.e., age, gender, marital status, labor sector, and work experience).

### 2.3. Procedure

The participants were recruited among psychology and labor relations students who had been previously trained to administer questionnaires. This procedure was performed pursuant to recommendations to apply this sampling technique [53]. Students contacted several educational centers to find professionals to participate in a cross-sectional survey. The participants received an explanation regarding the voluntary and confidential nature of their collaboration. All of them gave their consent to participate in the research. The complete process was conducted in accordance with the Declaration of Helsinki and the protocol was approved by the Ethics Committee of Jaume I University (UJI-A2018-10). The process was carried out during 2019.

### 2.4. Data Analysis

The data was analyzed using the SPSS software in its version 25.0 (SPSS Inc., Chicago, IL, USA). The first analysis consisted of descriptive statistics, including mean, standard deviation, and reliability of the study’s variables. After calculating the Pearson’s correlations among EI, burnout, and work performance, multiple mediation analyses were conducted, resulting in the proposed hypothesis (e.g., burnout has a moderation role between EI and work performance) in Figure 1. This procedure enabled the discovery of every EI dimension’s effects (predictor) through five different pathways for each EI branch and the total EI. An indirect path was statistically significant if the associated 95% confidence interval (CI; bias corrected) did not include zero. For this purpose, the macro PROCESS 3.3 [54] was applied. Following a bootstrap method with 10,000 samples of data, which generated 95% bias-corrected confidence intervals, it was possible to examine conditional models to predict direct and indirect effects between variables. To determine the relative magnitude of the specific indirect effects, contrasts were calculated using bias-corrected and accelerated bootstrap intervals. The effect of age, gender, and work experience was controlled to avoid a possible interference between EI and WP.

## 3. Results

### 3.1. Descriptive Analysis

The first analyses were designed to describe correlations, means, standard deviations, and reliabilities concerning the study variables (Table 2).

As shown in Table 2, EI correlated significantly with all burnout variables (emotional exhaustion: r = −0.22; cynicism: r = −0.29; professional efficacy: r = 0.41). In the same way, EI correlated with all work performance variables (task performance: r = 0.45; contextual performance: r = 0.37; counterproductive work behavior: r = −0.35), as well as with the global score called work performance (r = 0.39). As expected, exhaustion and cynicism correlated negatively with task and contextual performance, but positively with counterproductive work. Finally, professional efficacy correlated positively with task and contextual performance, but negatively to CWB, as expected. The findings showed good reliability of the study variables (between 0.79 and 0.91).

### 3.2. Multiple Mediation Analyses

**Figure 2:**
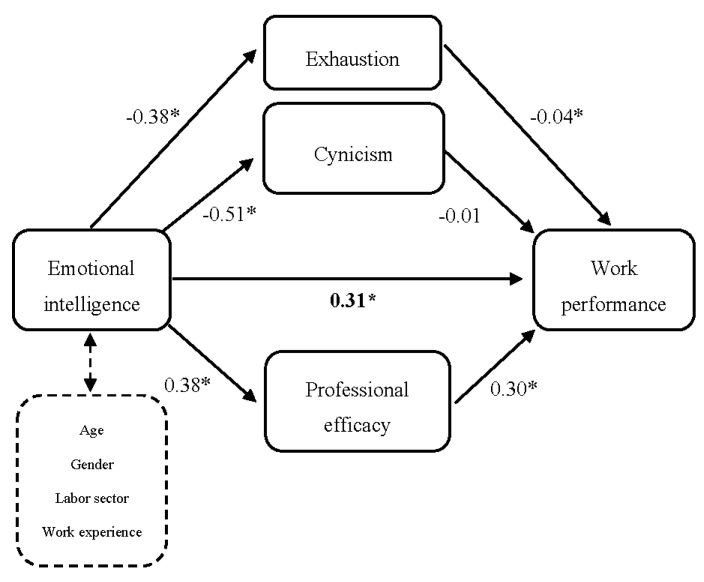
Mediation model of the burnout dimensions explaining the relationship between emotional intelligence and work performance. * *p* < 0.01.

A mediation analysis was performed to define the role of all three burnout dimensions. The confidence intervals (CIs) were established using a multiple mediator model. Table 3 shows the results of indirect effects as well as their 95% CIs. It is important to note that any covariable (age, gender, labor sector, work experience) had a significant effect. As seen in Figure 2, the bootstrap estimation showed the significant direct effect of EI on work performance (c = 0.31; *p* < 0.001). Once indirect effects (Table 3) were computed, exhaustion and professional efficacy showed a significant indirect effect (exhaustion indirect effect = 0.068; 95% CI = 0.01, 0.05; professional efficacy indirect effect = 0.181; 95% CI = 0.08, 0.17). However, cynicism did not show this significant effect (cynicism indirect effect = 0.038; 95% CI = −0.02, 0.06). Having examined the differences between the indirect effect of exhaustion and professional efficacy, it was found that professional efficacy had a stronger effect, which indicates that this dimension has greater importance as a mediator of the link between EI and work performance. In conclusion, once the effects of different covariables were controlled, professional efficacy and exhaustion completely mediated the relationship between EI and WP. The three burnout variables and covariables explained 39.1% of the variance in work performance (R^2^ adj = 0.39; *p* < 0.001). Post-hoc analyses were performed to discover the same moderation model for each EI branch. The results showed a similar pattern to the one reported earlier. Thus, each EI branch and its link to performance is fully mediated by professional efficacy and exhaustion, but not by cynicism.

## 4. Discussion

This research aimed to determine the mediator role of burnout in the relationship between EI and performance. In prior studies [19,20,21,22,23,24,25,26], EI has been independently tested, and results demonstrated that EI predicts a better performance. However, the current work offers a new perspective in understanding the role of burnout in this relationship.

Taking into account previous studies, it could be said that the ability to perceive, understand, use, and manage emotions is related to a better performance [27,31]. Our results support that EI could be a key personal resource to understand work performance, along with several variables, such as personality, intelligence, social support, etc. [2,6,55]. Through different mechanisms, EI allows people to face demanding situations and to better adapt to changing environments like modern workplaces [56]. Furthermore, our results indicate that all EI branches share the same relation with burnout and the WP dimensions, suggesting the all branches are of the same importance to explain the moderation mechanism. Therefore, EI performs a critical role in understanding workers’ outcomes.

It was also discovered that employees with high EI reported a lower level of exhaustion and cynicism, and a higher level of professional efficacy. These data are linked to previous findings which suggest that EI is negatively related to burnout in the workplace [42,43]. Workers with higher EI seem to be less affected by the negative consequences associated with burnout (i.e., cynicism, demotivation, distress, intention to quit, etc.), as well as by factors related to their negative attitudes in the workplace, thus experiencing an increase in their performance levels.

According to the data obtained for the purpose of exploring the mediator role of burnout in the relationship between EI and work performance, significant connections among EI, burnout, and WP were observed. The multiple mediation analyses showed that EI had an indirect effect on performance through professional efficacy and exhaustion—variables which fully mediated the link between EI and WP. These findings match with previous works that show the mediator effect of burnout between personal resources and work outcomes [42,45,46,57]. In this way, high EI levels help workers reduce their fatigue and improve their professional efficacy. Therefore, EI acts as a protective factor against the negative consequences of burnout and, in the process, increases workers’ performance. The mediator effect was significantly higher for professional efficacy. The positive effects associated with a good professional efficacy allow the worker to be more self-confident, proactive, and creative [58,59]. In conclusion, employees with a higher EI also scored higher in professional efficacy, which is related to a higher individual performance. Notwithstanding this, our data do not provide enough evidence to consider the dimension of cynicism as a mediator in the link to EI performance. Numerous studies have explored this relation. However, their results are inconclusive, hence the need for more research to understand this issue [60].

### 4.1. Limitations and Future Research

Several limitations suggest future lines of research for this work. First, it is important to underline that the cross-sectional data made it difficult to establish the direction of relationships between variables. The data was based on some extensive and robust scientific findings. Nevertheless, replicating these results with longitudinal methods might provide more information about EI’s contribution to individual job performance.

Second, some weakness is related to not having controlled the influence of factors like IQ or personality. It would have been advisable to take into account the different dimensions of personality, since it has been shown in previous studies that these have an influence on the results obtained in the workplace [61]. Specifically, taking into account the dimensions of Neuroticism and Responsibility would have been relevant, as their influence on professional success was observed [62].

Third, there is a limitation directly connected to how EI is measured, since the WLEIS is a self-report instrument. In addition, it is recommended to use both self-reports and performance tests to measure EI [12]. Therefore, in line with prior research examining predictive and incremental validity [63], an ability EI test such as MSCEIT [64] or MEIT [16] should be used. Yet, WLEIS is still one of the most used instruments to measure EI [65], and it has been designed specifically to assess the branches proposed by Mayer and Salovey’s model [16]. Furthermore, self-report questionnaires have some advantages, for instance, they can be administered and completed in less time [66].

Lastly, the sampling used may be problematic, since it was collected by means of a nonrandom technique—workers were selected through psychology students. Although this way to obtain data has demonstrated validity and reliability, as well as great utility in field studies within organizational psychology [52], this method may be biased toward more cooperative participants, thus limiting the generalization of the results.

### 4.2. Practical Implications

Despite these limitations, this work provides evidence of the important role EI and burnout play in explaining work performance. By developing their work and abilities, employees are the companies’ main assets to maintain and improve their competitiveness [21]. In fact, workplace health promotion programs have demonstrated a positive return on investment [67]. In the current global situation, every little bit can make a big difference, so a useful solution to reduce burnout impact is developing emotional skills [68,69]. For this reason, training programs should aim not only to develop the emotional abilities of professionals to prevent the problems of burnout, but also to promote individual outcomes. This approach could help to enhance emotion management, reducing the high impact that negative organizational and personal consequences—such as sick leaves or constant rotations—have on the company’s competitiveness [70,71].

## 5. Conclusions

In conclusion, these results underline the interactive role of EI and burnout as predictors of individual performance in a multioccupational sample of Spanish workers. Professionals with high EI have appropriate resources to deal with the demands of work and therefore minimize exhaustion. Moreover, EI also contributes to a higher professional efficacy, which helps maintain the perception of continued effectiveness at work and promote a better work performance. These findings demonstrate the importance of burnout in understanding work performance and emphasize the role of EI as a protective variable. This work supports previous research and reaffirms the advantage of developing these variables in companies. Therefore, it is necessary to develop and implement intervention programs in order to promote EI and create healthy workplaces which can prevent the development or chronic progression of burnout in employees while helping workers reach their best performance possible.

## Figures and Tables

**Table 1:** Individual characteristics of the study population.

Characteristics	
Age (Mean, SD)	38.1, 10.3
Gender	(%)
Men	41.4
Women	58.6
Marital status	(%)
Married	37.9
Single	33.7
In a relationship	20.3
Separated/divorced	5.1
Widowed	1.6
Occupational sector	(%)
Education	29.3
Healthcare	23.5
Industry	17.1
Hospitality and tourism	15.2
Commerce	7.8
Other sectors	7.1
Kind of employment	(%)
Private	50.6
Public	29.3
Self-employed	11.6
Unemployed	8.5
Work experience (Mean, SD)	11.89, 2.13
Organizational seniority (Mean, SD)	7.92, 1.94
Seniority in the job position (Mean, SD)	6.11, 1.74

Note: *N* = 1197.

**Table 2:** Descriptive Statistics and Correlations between Study Variables.

Variables	1	2	3	4	5	6	7	8
1. Emotional intelligence	-							
2. Exhaustion	−0.22 *	-						
3. Cynicism	−0.29 *	0.69 *	-					
4. Professional efficacy	0.41 *	−0.24 *	−0.34 *	-				
5. Task performance	0.45 *	−0.25 *	−0.27 *	0.54 *	-			
6. Contextual performance	0.37 *	−0.15 *	−0.20 *	0.58 *	0.41 *	-		
7. CWB	−0.35 *	0.50 *	0.49 *	−0.23 *	−0.20 *	−0.12 *	-	
8. Work performance	0.39 *	−0.28 *	−0.27 *	0.51 *	0.59 *	0.42 *	−0.35 *	-
Mean	5.55	2.44	1.80	4.76	3.12	3.03	1.61	3.10
Standard Deviation	0.84	1.50	1.53	0.80	0.60	0.68	0.90	0.72
α	0.91	0.90	0.86	0.79	0.80	0.85	0.81	0.88

Note: *N* = 1197. * *p* < 0.01. CWB = Counterproductive work behavior. α = Cronbach’s alpha.

**Table 3:** Multiple mediating analyses of burnout dimensions.

Model Pathways	Point Estimate	SE	Normal Theory Tests			95% Cias-Corrected CI	
			Effect	Z	*p*	Lower	Upper
Total effect	0.287	0.03				0.26	0.37
EI → E → WP	0.068	0.01	0.06	2.98	<0.01	0.01	0.05
EI → C → WP	0.038	0.01	0.03	2.54	0.09	−0.02	0.06
EI → PE → WP	0.181	0.02	0.18	−4.23	<0.01	0.08	0.17
Model 1: *p* < 0.01; R^2^ = 0.45; R^2^ adj = 0.39							
Total effect	0.199	0.03				0.23	0.36
SEA → E → WP	0.016	0.01	0.01	1.76	<0.01	0.01	0.04
SEA → C → WP	0.026	0.01	0.02	3.80	0.11	0.01	0.05
SEA → PE → WP	0.157	0.03	0.15	4.05	<0.01	0.11	0.22
Model 2: *p* < 0.01; R^2^ = 0.37; R^2^ adj = 0.24							
Total effect	0.275	0.04				0.45	0.29
OEA → E → WP	0.103	0.02	0.10	2.90	<0.01	0.15	0.26
OEA → C → WP	0.105	0.02	0.12	3.51	<0.05	0.17	0.29
OEA → PE → WP	0.067	0.02	0.06	1.92	<0.01	0.11	0.33
Model 3: *p* < 0.01; R^2^ = 0.36; R^2^ adj = 0.23							
Total effect	0.179	0.03				0.23	0.36
UOE → E → WP	0.016	0.01	0.01	1.26	<0.01	0.01	0.04
UOE → C → WP	0.026	0.02	0.02	3.71	0.07	−0.01	0.05
UOE → PE → WP	0.137	0.03	0.13	3.05	<0.01	0.06	0.12
Model 4: *p* < 0.01; R^2^ = 0.34; R^2^ adj = 0.25							
Total effect	0.195	0.04				0.45	0.29
ROE → E → WP	0.073	0.02	0.07	2.80	<0.01	0.15	0.36
ROE → C → WP	0.055	0.03	0.05	1.51	0.14	0.07	0.09
ROE→ PE → WP	0.067	0.01	0.06	1.02	<0.01	0.01	0.13
Model 5: *p* < 0.01; R^2^ = 0.26; R^2^ adj = 0.20							

Note: *N* = 1197. SE = Standard error. CI = Confidence interval. EI = Emotional intelligence. SEA = Self-emotion appraisal. OEA = Other-emotional appraisal. UOE = Use of emotions. ROE = Regulation of emotions. E = Exhaustion. C = Cynicism. PE = Professional efficacy. WP = Work performance.
